# The HeCz corpus: A large, richly annotated reading corpus of newspaper headlines in Czech

**DOI:** 10.3758/s13428-025-02863-4

**Published:** 2025-11-14

**Authors:** Jan Chromý, Markéta Ceháková, James Brand

**Affiliations:** 1 Faculty of Arts, Institute of Czech Language and Theory of Communication, Charles University, Prague, Czechia; 2 Department of Psychology, Edge Hill University, Ormskirk, United Kingdom

**Keywords:** Reading, Czech, Mood, Morphology, Sentence processing, Corpus

## Abstract

Large behavioral datasets that provide detailed data on reading processes are valuable resources for a range of researchers working in linguistics, psychology and cognitive science. This paper presents the HeCz corpus, which comprises self-paced reading data for 1919 newspaper headlines (23,634 words) in Czech, with each headline being accompanied by a yes–no comprehension question, resulting in a rich dataset of reading times for each individual word and comprehension accuracy. The corpus is novel in terms of the sheer scale of data collection, with 1872 native Czech speakers, each reading approximately 120 headlines, with 1162 of those participants also completing the experiment again in a re-testing session using the same stimuli approximately 1 month later. There is participant level meta-data also available relating to basic demographic information, reading habits and a profile of their mood state prior to completing the experiment. Beyond the behavioral and demographic data, we also include a range of linguistic annotations for several variables, e.g., frequency, surprisal, morphological tagging. To better understand how these variables might impact processing, we present exploratory analyses where we predicted the reading times for words, with the results indicating important roles for linguistic, demographic, and methodological variables. Given the range of multidisciplinary applications of the HeCz corpus, we hope that it will provide a valuable and unprecedented resource for a range of research applications related to reading processes.

## Introduction

Language corpora are collections of texts used in linguistic research (McEnery and Hardie, 2011). They vary in size, ranging from small corpora with tens of thousands of tokens (Almut, 2010) to very large with billions of tokens, such as GloWbE (Davies and Fuchs, 2015). Corpora also differ in the languages they target (monolingual vs. multilingual/parallel), the types of texts they include (e.g., news, scientific articles, transcripts of spoken interactions, texts by specific authors, or those produced by L2 speakers), their annotations (e.g., segmentation, lemmatization, morphological or syntactic annotation, or other types), and modality (written, spoken, or multimodal) (cf. McEnery and Hardie, 2011). These features make corpora invaluable tools for a wide range of scientific inquiries.

For nearly six decades, corpora have played a crucial role in studying language, offering insights into language use (cf. Kučera and Francis, 1967; McEnery and Hardie, 2013). Traditional corpora focus on textual data, allowing researchers to study language production through its end products (e.g., written texts or transcriptions of spoken communication). However, they typically lack behavioral data reflecting how texts are processed during comprehension.

Recently, researchers have developed corpora that combine annotated texts with behavioral measures, such as eye-tracking, self-paced reading or even EEG data, to capture how individuals process language during reading (e.g., Frank et al. , 2013; Futrell et al., 2021; Siegelman et al. , 2022; Luke and Christianson, 2018; Kliegl et al., 2006; Laurinavichyute et al., 2019; Pan et al., 2022; Yan et al., 2014; Özkan et al., 2021; Hollenstein et al., 2018,, 2023; Frank and Aumeistere, 2024; Kuperman et al., 2025; Jakobi et al., 2025; Berzak et al., 2022). These corpora enable researchers not only to examine how language is used but also to investigate real-time language processing.

Existing behavioral corpora differ not only in the technique used for data collection (e.g., eye-tracking, self-paced reading, EEG), but also in several other aspects. Some corpora comprise longer text passages typically sourced from existing documents, such as Provo (Luke and Christianson, 2018), MECO (Siegelman et al., 2022; Kuperman et al., 2023), or PoTeC (Jakobi et al., 2025), or MultiplEYE (Jakobi et al., 2025). While others focus on the processing of isolated sentences, such as the UCL corpus (Frank et al., 2013), the Russian Sentence Corpus (Laurinavichyute et al., 2019), CELER (Berzak et al., 2022), or RaCCooNS (Frank and Aumeistere, 2024).

Annotation is another key source of variation within the available datasets, with different behavioral corpora typically containing different types of linguistic annotation, including morphological or syntactic information (e.g., Futrell et al., 2021; Berzak et al., 2022; Jakobi et al., 2025; Laurinavichyute et al., 2019). Many also include external annotations, such as predictability or surprisal values (e.g., Frank et al., 2013; Berzak et al., 2022; Frank and Aumeistere, 2024; Laurinavichyute et al., 2019; Jakobi et al., 2025) frequency measures (e.g., Jakobi et al., 2025; Futrell et al., 2021) or sentiment scores (Hollenstein et al., 2018). These can prove valuable resources for extending potential analyses of the data towards exploring various processing phenomena (e.g., Meister et al., 2024).

Additionally, there are sources of variation that relate specifically to the participants being sampled for the collection of the data being made available in the corpora. Some datasets have focused exclusively on L1 speakers of a language (e.g., Futrell et al., 2021; Luke and Christianson, 2018; Frank et al., 2013; Frank and Aumeistere, 2024), while others also include L2 speakers (e.g., Berzak et al., 2022; Kuperman et al., 2023; Jakobi et al., 2025), or target specific populations, such as expert vs. non-expert readers (Jakobi et al., 2025). This has allowed the use of these datasets to facilitate research on language processing and comprehension, where specific questions can be investigated that relate directly to the participant level information (e.g., Landwehr et al., 2025). Nevertheless, the types of participant information that is likely to be considered important when collecting data for these corpora will naturally depend on the primary aims outlined by the researchers. For instance, minimal demographic information is provided in some corpora (e.g., Futrell et al., 2021; Luke and Christianson, 2018) while others include basic demographic details such as age and gender (e.g., Frank and Aumeistere, 2024; Laurinavichyute et al., 2019: Jakobi et al., 2025). Contrastingly, there are instances where highly detailed participant information has been collected to allow for a more nuanced understanding of the data to be gained, for example by also including L2 proficiency scores (e.g., Berzak et al., 2022; Kuperman et al., 2023; Hollenstein et al., 2018), or even a broader set of psychometric test results (Jakobi et al., 2025; Siegelman et al., 2022).

Importantly, existing behavioral corpora have notable limitations in size, both in terms of the linguistic units they contain and the number of participants included. These constraints are understandable, given the demanding nature of data collection, especially for techniques like eye-tracking or EEG. For instance, the Natural Stories Corpus (Futrell et al., 2021) comprises 485 sentences (10,245 word tokens) read by 181 native English speakers using self-paced reading, resulting in 848,768 observations. Similarly, the self-paced reading component of the UCL Corpus (Frank et al., 2013) includes 361 English sentences read by 117 participants, totaling 274,893 observations. Eye-tracking corpora tend to be even smaller, but have substantially higher data resolution. The Potsdam Sentence Corpus (Kliegl et al., 2006) contains 144 German sentences (1138 words) read by 222 participants, while the Provo Corpus (Luke and Christianson, 2018) features 55 short texts (2689 words) read by 84 native English speakers. A multilingual example is the MECO corpus (Siegelman et al., 2022; Kuperman et al., 2023; Siegelman et al., 2025), which includes L1 reading data for 21 languages. The materials are 12 Wikipedia-style encyclopedic entries (each approximately 8–12 sentences long), five of which were translations from English, which allows for direct comparisons of reading data between the languages.

Despite their size limitations, behavioral corpora are highly informative for a variety of research purposes. For example, the cross-linguistic analysis conducted with the MECO corpus (Siegelman et al., 2022) revealed that readers across different languages exhibit similar fixation times during reading but differ in their word-skipping rates. Based on the Provo Corpus, Luke and Christianson (2016) found that reading is more strongly facilitated by general semantic and morphosyntactic predictability than by full word-form predictability. Similarly, Kennedy et al. (2013) used the Dundee Corpus to demonstrate that foveal fixation durations are influenced by the predictability of parafoveal words, underscoring the importance of parafoveal processing in reading.

Behavioral corpus data also serve as important benchmarks for other studies using similar techniques. For instance, experimental stimuli can be derived from these corpora, or their data can be used to compare results across different methodologies. Wehbe et al. (2021), for example, utilized data from the Natural Stories Corpus to compare self-paced reading and fMRI data.

In this paper, we present HeCz, a large-scale behavioral corpus of Czech newspaper headlines. The main strength of the corpus lies in its size – the participant sample includes more than 1800 L1 Czech speakers, and the textual data comprise over 23,000 tokens – making it, to our knowledge, the largest openly available behavioral corpus focused on L1 processing. The HeCz corpus is also unique in that approximately two-thirds of the participants were tested again after a 1-month interval, enabling large-scale analyses of long-term task adaptation.

Like several other corpora, HeCz offers rich linguistic annotations for each word token, including morphological information (similarly to the Russian Sentence Corpus, see Laurinavichyute et al. , 2019), frequency measures, and surprisal values. The corpus also includes basic demographic information, along with previously under-explored assessments, such as participants’ reading habits (see Section “Reading habits”) and Profile of Mood States scores (McNair et al., 1971).

The HeCz corpus is also distinctive in terms of the type of textual material it uses: online newspaper headlines. This choice was motivated by several considerations. First, online newspaper headlines constitute relatively short, self-contained linguistic units – typically one or two sentences – that are commonly read in isolation, without preceding context and often without continued reading of the full article. Moreover, they are written with this stand-alone function in mind. Given our aim to avoid longer continuous texts, we believe that headlines offer greater ecological validity than artificially constructed sentences or decontextualized excerpts from longer texts. They reflect naturally occurring language that is both accessible to readers and functionally suited to isolated presentation in experimental settings.

## Self-paced reading

As follows from the Introduction, most existing behavioral corpora have used eye-tracking as the primary data collection technique. Eye-tracking (cf. Rayner, 1998) provides highly detailed insights into reading behavior and offers strong ecological validity. However, it also comes with notable disadvantages – chiefly the time-intensive nature of data collection, which limits the size of the datasets that can be reasonably obtained (see size limitations of existing corpora mentioned in the Introduction).

For the HeCz corpus, we opted instead for self-paced reading (SPR), which allowed us to collect an unprecedented amount of reading data within a relatively short time frame. In other words, we prioritized scalability and coverage over the fine-grained precision afforded by eye-tracking.

Despite its limitations, SPR remains a standard and widely used method in language processing research (Just and Carpenter, 1980; Jegerski, 2013; Chromý and Dotlačil, 2022). In this paradigm, participants read linguistic material (typically sentences or short texts) incrementally by pressing a button. In the most common implementation, the first button press reveals the first word of the stimulus, and subsequent presses reveal each new word while hiding the previous one. Reaction times for each word are recorded and interpreted as reflecting processing difficulty (Just and Carpenter, 1980; Ferreira and Henderson, 1990).

Compared to natural reading as measured by eye-tracking, SPR does not allow for regressions (though see Paape and Vasishth, 2022), and effects often appear not on the target word but on subsequent words (spillover effects; see Boyce et al., 2020). Moreover, comprehension accuracy tends to be slightly lower in SPR than in full-sentence reading, suggesting that the task is cognitively more demanding. Nevertheless, the difference is typically quite negligible with only very small differences being reported in experimental studies (e.g., Ceháková and Chromý, 2023).

Overall, SPR remains widely used due to its methodological simplicity, low cost, and suitability for large-scale, web-based data collection.

**Table 1:** Main demographic characteristics, self-reported reading difficulties, alcohol consumption, and noise levels of the participant samples in each data collection round

Round	*N*	Gender	Age (years)	Reading	Alcohol	Noise level
				problems	consumption	
					(24h/current)	
Round 1	1872	1485 F, 368 M, 16 nonbinary, 3 undisclosed	22.9 (SD = 5.5)	63 (3.4%)	236/9 (12.6%/0.5%)	2.6 (SD=1.4)
Round 2	1162	918 F, 233 M, 8 nonbinary, 3 undisclosed	23.3 (SD = 6)	28 (2.4%)	85/5 (7.3%/0.4%)	2.7 (SD=1.4)

See Section “Demographic questionnaire” for details on the wording of the questions

## Structure of the Czech language

Czech is a West Slavic language with several typological features that distinguish it from languages such as English, German, or Spanish. It has a relatively transparent orthography, with a near 1:1 phoneme–grapheme correspondence (cf. Caravolas, 2004) and uses the Latin alphabet. Morphologically, Czech is highly inflectional, with a complex system of endings. The endings of nouns, adjectives, and most pronouns and numerals carry the information about gender (feminine, masculine, neuter), case (nominative, genitive, dative, accusative, vocative, locative, instrumental), and number (singular, plural). Verbs exhibit rich inflection as well, with endings reflecting tense, voice, person, number, and mood (cf.  Short, 2018). Czech also has relatively free word order, though there is a general preference for SVO structures (Šimík and Wierzba , 2017; Siewierska and Uhliřová, 2010).

## The HeCz corpus

In this section, we provide a brief overview of the HeCz corpus, including its features and availability. Details regarding data collection, stimulus annotation, and related procedures are discussed in the subsequent sections.

The HeCz corpus comprises four main data sources:

1. *Textual data*: The corpus includes 1919 newspaper headlines (totaling 23,634 tokens) sourced from Seznamzpravy.cz, one of the most widely read online news portals in the Czech Republic. The textual data are fully lemmatized and morphologically annotated.
2. *Participant demographics and mood assessment*: A total of 1872 native Czech speakers participated in the study. Each participant completed a brief demographic questionnaire and reported their current mood prior to the reading task. Mood was assessed using the Czech adaptation of the Profile of Mood States questionnaire (Stuchlíková et al., 2005; McNair et al., 1971).
3. *Reading data*: Reading data were collected over two rounds, approximately 1 month apart. In the first round, all 1872 participants completed the reading task. In the second round, a subset of 1162 participants was re-tested using the same stimuli. The corpus includes a total of 4,840,075 observations, representing individual reaction times for each word in the reading task.
4. *Comprehension accuracy*: After reading each headline, participants answered a yes–no comprehension question. These questions were designed to capture a variety of information types to ensure a comprehensive evaluation of participant understanding.

## Participant-level data

Before the reading task, participants were given a demographic questionnaire (available with English translation on the project’s OSF website: https://osf.io/jg8ha/) and they completed the questionnaire from the Profile of Mood States (Stuchlíková et al., 2005). All participants were Charles University students and were awarded with a course credit for taking part in the data collection.

### Demographic questionnaire

All participants completed a basic demographic questionnaire at the beginning of the experiment. A summary of this information is provided in Table 1.

To account for potential confounds relating to participant or environmental level variation, several control questions were included. One question asked whether participants experience any reading difficulties such as dyslexia. Two further questions targeted recent alcohol consumption (for ethical reasons, these two questions were voluntary): (i) Did you drink alcohol during the last 24 h (more than one glass beer/wine)? (ii) Are you now under the influence of alcohol? Finally, as the data collection was web-based, we also asked participants to rate the noisiness of their environment on a seven-point scale (1 = very quiet, 7 = very noisy).

These control questions are important for assessing data quality. While no participants were excluded based on their responses, we encourage users of the corpus to consider filtering based on these variables, depending on their research goals. A summary of responses to all these questions is presented in Table 1.

#### L2 proficiency and exposure

The HeCz corpus comprises data on first-language (L1) processing. However, we also collected data on participants’ second-language (L2) proficiency and exposure. By “second language,” we explicitly referred to the foreign or second language that participants were most proficient in. Since these measures were and our procedure was already time-consuming, we opted for a simple self-assessment measure instead of more complex questionnaires such as the LEAP-Q (Marian et al., 2007). Participants reported their L2 proficiency on the six-point CEFR scale (A1–C2), where A1 represents a complete beginner and C2 corresponds to a highly proficient user who understands everything with ease and can express themselves very fluently and precisely (Council of Europe, 2001). This scale is well known in Czechia, and participants are generally familiar with their level due to various exams they have to take within their studies. The option “I don’t know” was not provided for this question, in order to encourage participants to make an informed self-assessment. Table 2 shows the distribution of L2 proficiency across the two rounds.

**Table 2:** Self-reported L2 proficiency in the two data collection rounds

Round	A1	A2	B1	B2	C1	C2
Round 1	29 (1.5%)	52 (2.8%)	337 (18%)	816 (43.6%)	523 (27.9%)	115 (6.1%)
Round 2	20 (1.7%)	45 (3.9%)	216 (18.6%)	505 (43.5%)	303 (26.1%)	73 (6.3%)

Each column represents a point on the CEFR scale (A1–C2), where A1 indicates a complete beginner and C2 corresponds to a highly proficient user who understands everything with ease and can express themselves very fluently and precisely. The numbers indicate the absolute number of participants who selected each level, with percentages calculated separately for each round

Additionally, participants self-assessed their exposure to foreign languages using a scale from 0 (“not at all”) to 10 (“all the time”). The average exposure score in the first round was 5.94 (SD = 2.6), and in the second round, it was 5.96 (SD = 2.6).

**Fig. 1:**
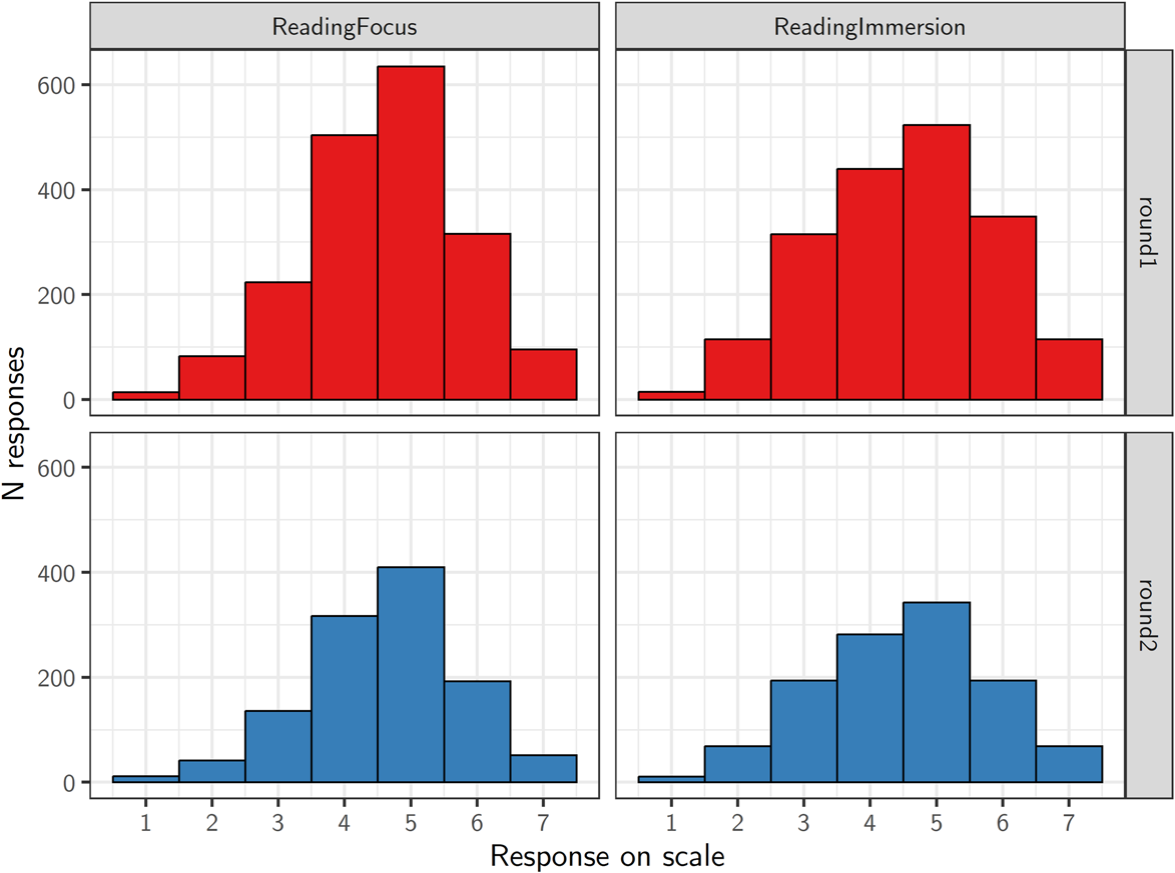
Distribution of responses to the two questions targeting reading habits across the two data collection rounds. The wording of each question is provided in Section “Reading habits”

#### Reading habits

It has been documented that readers may differ substantially in their reading speed (cf. Traxler et al., 2012) which appears to be at least partly related to the depth of processing (Mak and Willems, 2019; Ferreira and Yang, 2019). Therefore, our aim was also to assess individual differences in participants’ typical reading styles. Since, to our knowledge, there is no standardized questionnaire targeting these issues, we developed two questions assessing participants’ reading habits. The questions used seven-point scales and focused on (i) the extent to which participants pay attention to how the text is formulated, and (ii) the extent to which they immerse themselves in various details of a given text. The English translations of the two questions are presented below.

1. **Reading focus**: While reading, to what extent do you focus on how the text is written and how individual formulations change its meaning? (1 = not at all, 7 = all the time)
2. **Reading immersion**: While reading a text, I am typically immersed in all of its details and I am trying to understand it fully. (1 = absolutely disagree, 7 = absolutely agree)

The distribution of the answers to these two questions in the two rounds is presented in Fig. 1.

### Profile of Mood States

**Fig. 2:**
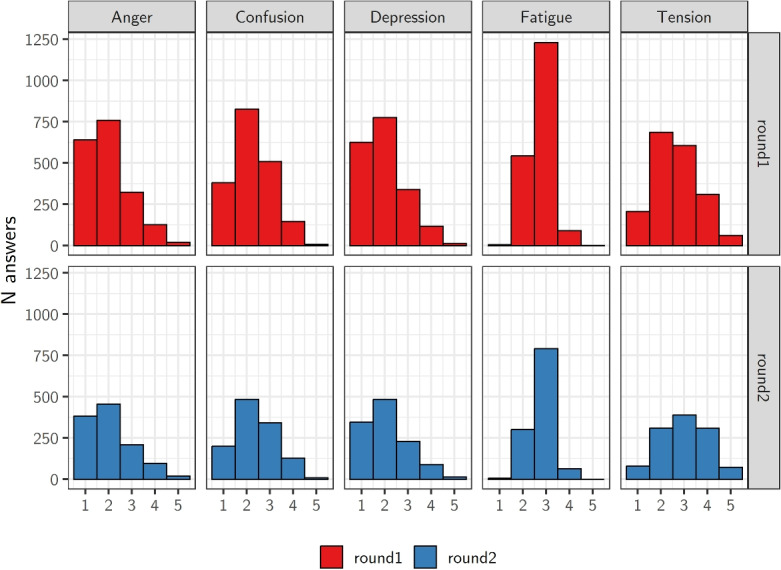
Distribution of participants’ scores for the five dimensions of the Profile of Mood States (anger, confusion, depression, fatigue, tension) across the two data collection rounds

After completing the initial questionnaire, participants were presented the Czech adaptation of the Profile of Mood States (Stuchlíková et al., 2005), a standardized tool for assessing mood across five dimensions: anger, confusion, depression, fatigue, and tension. Participants were presented 32 adjectives (e.g., “exhausted”) and asked to indicate the extent to which each applied to their state during the past week, including the day of data collection. The responses were recorded on a five-point scale ranging from 1 – “not at all” to 5 – “extremely.” The scores for adjectives associated with each mood dimension were averaged and are included in the corpus as measures of the corresponding mood state. The distributions of participants’ scores are presented in Fig. 2.

The inclusion of mood-related data in the HeCz corpus was motivated by previous research suggesting that affective states can influence language processing (e.g., Egidi and Nusbaum, 2012; Matovic et al., 2014; Van Berkum et al., 2013). Although this connection is less established than other typically examined effects (such as lexical frequency), we considered it worthwhile to explore. The Czech adaptation of the Profile of Mood States Questionnaire was readily available and brief to administer, making it feasible to include without increasing participant burden. By collecting this data, we aimed to expand the utility of our corpus for future research on how individual affective states may modulate sentence comprehension in real time (cf. Chwilla, 2022).

## Textual data

### Materials

We randomly selected 2500 headlines from Seznamzpravy.cz, one of the most widely read online news portals in Czechia. The headlines were all from the year 2019, making them relatively distant from the time of data collection (2023). This time period was also chosen to avoid the major and recurring topic of COVID-19. Each headline was manually reviewed, and those that were incomplete sentences or lacked coherence (e.g., “Czech media under pressure, spitting on the cameraman and an Oscar for the Czech Republic”) were excluded.

Next, yes–no comprehension questions were created for each headline, targeting a variety of syntactic positions (e.g., subject, object, locative adjunct) to discourage participants from focusing exclusively on specific parts of the sentences (cf. Chromý and Tomaschek, 2024). Headlines for which meaningful comprehension questions could not be devised were also excluded.

After this filtering process, the final corpus included 1919 headlines (23,634 word tokens). The average headline length was 12.3 words (SD = 2.71), with an average word length of 5.53 characters (SD = 2.8). An example headline with a following comprehension question is presented in (1).

1. Headline: Už brzy se vypije víc českého piva v cizině než u nás, říká šéf Staropramenu Question: Řekl to tiskový mluvčí Staropramenu? English translation: ‘Soon more Czech beer will be drunk abroad than here, says the head of Staropramen’ —‘Did the Staropramen spokesperson say that?’

### Annotation

Stimuli annotation was done using openly available tools for Czech. These tools are summarized in Table 3.

**Table 3:** Overview of stimulus annotation and the tools applied

Tool	Type of annotation	Source
MorphoDiTa: Morphological Dictionary and Tagger	lemmatization, morphological tagging (part of speech, and additional morphological details relevant to its category, e.g., case, number, and gender for nouns; voice, aspect, person, and tense for verbs)	(Straková et al., 2014)
SYN2020 corpus	corpus frequencies	(Křen et al., 2020)
CzeGPT-2 generative transformer model	surprisal values	(Hájek and Horák, 2024)
R	word length, headline length	(R Core Team, 2024)
manual annotation	multiword sequences	–

The final set of headlines was automatically lemmatized and morphologically annotated using MorphoDiTa: Morphological Dictionary and Tagger (Straková et al., 2014). Both lemmatization and morphological tagging were manually checked and corrected by two independent coders (L1 speakers of Czech with advanced linguistic training) to ensure accuracy. Each word token in the corpus is annotated with its lemma, part of speech, and additional morphological details relevant to its category (e.g., case, number, and gender for nouns; voice, aspect, person, and tense for verbs).

In addition to this, multiword sequences were manually identified and flagged (e.g., *Premier League*, *Pulp Fiction*, *Nord Stream 2*, *SUV model Y*, *Amnesty International*, etc.). This annotation is significant for analysis as such sequences often behave differently from individual words, and their components may not correspond to existing standalone words in Czech.

The HeCz corpus also includes frequency data extracted from the SYN2020 corpus, a representative written corpus of Czech (Křen et al., 2020). The frequency annotation encompasses: (i) absolute frequencies for both lemmas and individual word forms, (ii) relative frequencies (occurrences per 1,000,000 words), (iii) the number of documents in which each lemma or word form appears, (iv) averaged reduced frequencies (Savický and Hlaváčová , 2002), which is an adjusted frequency metric accounting for lemma or word dispersion across the corpus, and (v) Zipf values for both lemmas and individual word forms. The Zipf values were calculated following Van Heuven et al. (2014), i.e., all frequency values were first adjusted using Laplace transformation so that frequencies of 0 would be avoided (cf. Brysbaert and Diependaele, 2013), the relative frequency was calculated as frequency per million words while the total corpus size was increased by the number of unobserved word types (i.e., words which have 0 frequency in SYN2020). The Zipf value was then calculated as log10 (frequency per million words) + 3.

The annotation also includes surprisal estimates for individual words in each sentence. Surprisal is defined as the negative log-probability of a word given its preceding context (Levy, 2008; Hale, 2001). More recently, large language models are commonly used to derive surprisal estimates, as they are trained to generate probability distributions over tokens – distributions that often align closely with the predictive mechanisms observed in human sentence processing (Goldstein et al., 2022; Schrimpf et al., 2021; Goodkind and Bicknell, 2018; Shain et al., 2024; Wilcox et al., 2023). However, several studies have also highlighted cases where the correlation between model-derived surprisal and human processing strategies is limited or variable (Huang et al., 2024; Bolliger et al., 2024; Kuribayashi et al., 2021; Škrjanec et al., 2023).

We used the surprisal package in Python (Sathe, 2025) to calculate the values and employed a publicly available CzeGPT-2 generative transformer model (Hájek and Horák, 2024), which has 124 million trainable parameters and was trained on 5GB of Czech documents originating from the internet. Surprisal for each word was calculated as the sum of the surprisals of its constituent sub-word tokens (cf. Bolliger et al., 2024).

Finally, the annotation includes details on word length (in characters) and headline length (in words).

## Behavioral data

### Procedure

The self-paced reading task was web-based: programmed in JsPsych v7.0.0 (De Leeuw, 2015) and run via JATOS (Lange et al., 2015). Data were collected during early April and late May 2023.

The headlines were divided into 16 random lists, each containing 119 or 120 headlines. The average headline length in each of the lists was between 12.5 and 13.5 words. Before the reading task, participants were randomly assigned one of these lists. Participants who took part in both rounds were ascribed to the same list each time. The order of stimuli was randomized for each individual participant in each round. On average, we had 117 participants per list in the first round (range = 108–141 participants) and 72.6 in the second round (range = 61–86 participants).

**Fig. 3:**
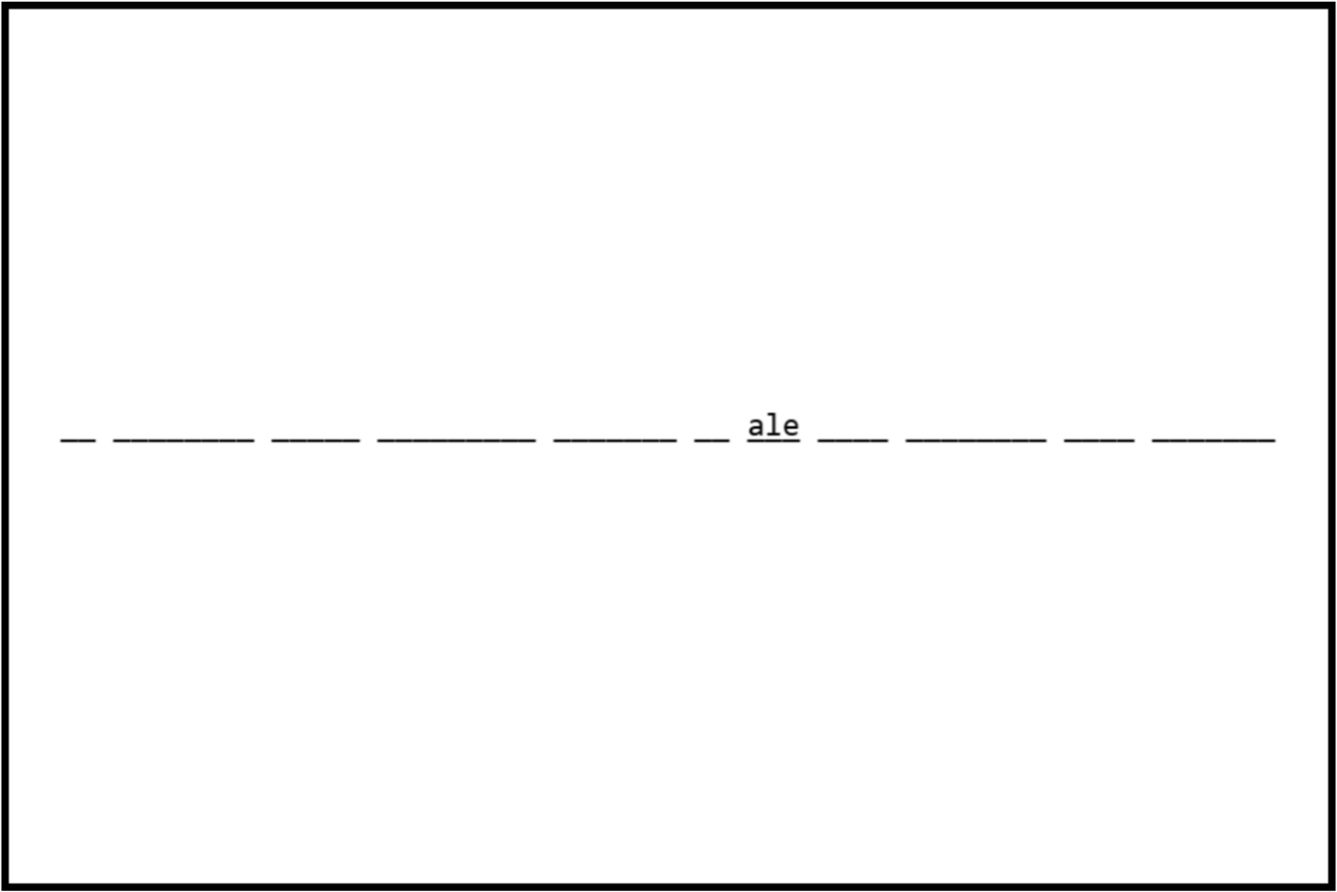
Screenshot from the experiment showing an example of the moving-window self-paced reading presentation

**Fig. 4:**
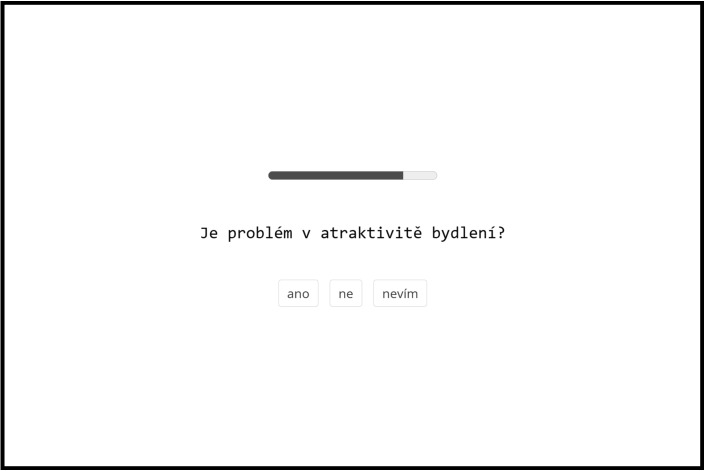
Screenshot from the experiment illustrating the presentation of a yes/no question, with a progress bar shown above

We used a moving-window self-paced reading presentation where the sentences were presented as a series of underscores and each button press revealed one word and hid the previous one. After reading each headline, a comprehension question was presented on a separate screen, requiring a ‘yes’, ‘no’, or ‘I don’t know’ response. Figures 3 and 4 present screenshots of the reading and answering procedures.

### Reaction times

Reaction times were automatically recorded for each word in milliseconds. No data trimming was performed to enable corpus users to trim the data according to the principles they prefer.

**Table 4:** Distribution of the main parts of speech in the HeCz corpus of newspaper headlines and in the corpus SYN2020 (Křen et al., 2020)

POS	N HeCz	% HeCz	N SYN2020	% SYN2020	% difference
adjective	2520	10.95	10,887,432	11.06	0.11
adverb	905	3.93	7,130,197	7.24	3.31
conjunction	753	3.27	8450,009	8.58	5.31
noun	9398	40.83	28,840,112	29.30	-11.53
numeral	795	3.45	2,918,031	2.96	-0.49
preposition	3029	13.16	10,702,152	10.87	-2.29
pronoun	1265	5.50	11,396,923	11.58	6.08
verb	4352	18.91	18,108,770	18.40	-0.51

**Table 5:** Basic descriptive statistics for the main parts of speech

POS	*N*	Word	Lemma	Zipf	Word	Zipf	Surprisal	RT	RT
		Length	Relative	Value	Relative	Value		Round 1	Round 2
			Frequency	(lemma)	Frequency	(Word)			
adjective	2520	8.52	50.29	4.01	17.68	3.51	12.45	446.56	404.02
	(10.95%)	[8.41;	[42.41;	[3.96;	[15.52;	[3.47;	[12.09;	[405;	[447.64;
		8.64]	59.24]	4.06]	20.04]	3.56]	12.82]	445.49]	405.98]
adverb	905	6.34	254.19	4.75	213.05	4.69	7.4	415.18	379.1
	(3.93%)	[6.1;	[194.25;	[4.63;	[161.37;	[4.58;	[7.08;	[380.39;	[416.71;
		6.6]	321.58]	4.87]	268.93]	4.8]	7.73]	413.8]	381.72]
conjunction	753	3.73	3030.17	6	2778.57	5.94	3.36	396	362.45
	(3.27%)	[3.2;	[1328.77;	[5.74;	[1214.02;	[5.69;	[3.12;	[363.63;	[397.24;
		4.27]	5591.97]	6.26]	5287.33]	6.18]	3.61]	394.73]	364.82]
noun	9398	7.11	49.36	4.03	20.97	3.69	9.55	448.61	405.64
	(40.83%)	[7.05;	[45.35;	[4;	[19.79;	[3.66;	[9.39;	[406.12;	[449.16;
		7.16]	53.65]	4.06]	22.29]	3.71]	9.7]	448.08]	406.61]
numeral	795	4.27	108.2	4.01	81.93	4.1	7.23	413.16	374.27
	(3.45%)	[4;	[76.89;	[3.81;	[65.52;	[3.94;	[6.87;	[375.73;	[414.85;
		4.56]	146.44]	4.19]	98.71]	4.25]	7.62]	411.57]	
preposition	3029	4.04	2443.7	5.59	2601.16	5.66	4.02	389.73	359.7
	(13.16%)	[3.48;	[1234.82;	[5.25;	[1309.49;	[5.4;	[3.91;	[360.31;	[390.38;
		4.64]	3944.18]	5.89]	4198.59]	5.93]	4.12]	389.08]	360.93]
pronoun	1265	4.34	3172.63	5.89	820.13	5.39	3.96	390.17	359.03
	(5.5%)	[4.05;	[1404.4;	[5.46;	[467.75;	[5.28;	[3.76;	[359.96;	[391.21;
		4.61]	5389.24]	6.23]	1268.81]	5.5]	4.17]	389.14]	360.95]
verb	4352	7.29	114.85	4.31	40.79	3.7	8.75	432.48	391.81
	(18.91%)	[7.21;	[66.66;	[4.27;	[29.71;	[3.66;	[8.58;	[392.46;	[433.19;
		7.37]	195.99]	4.35]	55.21]	3.74]	8.92]	431.77]	393.09]

*N* represents the number of occurrences. For each other variable, the mean is followed by 95% confidence intervals estimated using a non-parametric bootstrap with 2000 resamples. Word length is measured in characters. Lemma relative frequency and word relative frequency are measured as the number of occurrences per 1,000,000 tokens in the SYN2020 corpus (Křen et al., 2020). Surprisal values are estimated using the CzeGPT-2 generative transformer model (Hájek and Horák, 2024). Reaction times are reported separately for each data collection round and are based only on the subset of participants who took part in both rounds, consistent with the analysis presented in Section “Uses of the corpus”

### Response data

The corpus includes seven types of yes–no comprehension questions, each designed to target specific types of information: (i) adverbials, (ii) attributes, (iii) locations, (iv) patients, (v) agents, (vi) temporal information, and (vii) verbs.

Each question was annotated with its correct answer, and participants’ responses were coded for accuracy (correct or incorrect). During data processing, we filtered out all questions with an overall accuracy below 65% (*N* = 102). We then manually checked whether the correct answer had been coded correctly and whether the question was unambiguous – that is, whether it had a clearly correct response. Thirty-seven questions were identified as potentially misleading, as they lacked a clearly correct answer (typically, these were questions with a literal incorrect answer but a potentially correct answer if certain inferences were made). While the corresponding response data were retained in the corpus, the correct answer for these items was replaced with a missing value (NA), and participants’ responses were not coded for accuracy. Participants’ average response accuracy in the first round was 89.1% (with SD = 3.11%) and in the second round, it was 90.1% (SD = 2.99%).

In addition to accuracy, the data includes response times (measured in milliseconds), representing the interval between the start of the question’s presentation and the participant’s response. The corpus also provides information on the position of the targeted linguistic element within the headline (the number of the word in the given word sequence).

## Uses of the corpus

The HeCz corpus is by far the largest reading corpus in the world to date. Moreover, it contains morphological annotation which enables types of analyses which have not been yet done on samples of this size.

The HeCz corpus thus offers diverse applications for researchers across various fields. It enables the investigation of linguistic factors such as surprisal, frequency, word length, morphological complexity, and their effects on reading and language processing. Additionally, the corpus provides a unique resource for studying language processing in a morphologically complex language like Czech.

Since approximately two-thirds of the participant sample completed the same reading task twice, with an interval of about 1 month between sessions, the corpus provides a unique data source for studying task adaptation in language processing (cf. Prasad and Linzen, 2021; Chromý and Tomaschek, 2024).

The corpus also facilitates research into the relationship between mood and language processing, as well as inter- and intra-individual differences in reading behavior and comprehension. Moreover, it serves as a valuable tool for exploring the self-paced reading method itself, offering insights into its strengths and limitations as a technique in behavioral research.

Finally, the HeCz corpus is highly suitable for computational modeling of language comprehension, integrating textual and behavioral data to enhance our understanding of real-time language processing.

### Descriptive statistics for parts of speech

To assess general textual differences between the corpus of newspaper headlines and written Czech more broadly, we compared the distribution of parts of speech in the HeCz corpus with their distribution in the balanced corpus of written Czech, SYN2020 (Křen et al., 2020). The results are presented in Table 4. We found very similar proportions of adjectives, verbs, and numerals (differences of less than 1%), slightly lower usage of adverbs, and slightly higher usage of prepositions in the HeCz corpus. The most notable difference was observed for nouns: in SYN2020, nouns accounted for 29.29% of these basic parts of speech, whereas in the HeCz corpus they made up 40.83%.

Overall, headlines in the HeCz corpus appear more nominal in nature, with fewer functional words that establish textual cohesion (such as pronouns and conjunctions). This likely reflects the fact that headlines are short, often self-contained texts, which offer limited opportunities for the use of connectives or anaphoric reference. The higher use of nouns may thus compensate for the reduced use of pronouns. Notably, the similar proportion of verbs in both corpora supports the sentence-like character of the headlines in HeCz–an observation that aligns with previous findings on online news headlines (cf. Nickl et al., 2025).

To provide an overview of the key lexical and processing characteristics of the data, Table 5 presents mean values of word length, lemma, and word form relative frequency, surprisal, and reaction times (RTs) for the main parts of speech in the corpus, separately for each data collection round. The table also includes the number of occurrences for each part of speech. These descriptive statistics offer insight into the typical properties of the stimuli and participant responses across lexical categories. They also serve as a basis for interpreting and contextualizing the results of the statistical models reported in later sections.

### Exploratory findings

The HeCz corpus has already been utilized for a preliminary analysis investigating the effects of stimulus order, headline length, and data collection round on both reaction times (RTs) and response accuracy (Chromý et al., 2023). Results indicated that participants exhibited a gradual increase in reading speed as the experiment progressed, similarly to other studies (e.g., Prasad and Linzen, 2021; Chromý and Tomaschek, 2024). Additionally, participants were both faster and more accurate during the second round of testing, suggesting a persistent task adaptation lasting at least 1 month. The analysis further revealed that headline length significantly affected both response accuracy and reaction times: longer headlines were associated with lower accuracy but faster RTs for individual words.

In this paper, we conducted two exploratory analyses. The first examined the effects of word surprisal, Zipf value for lemmas, Zipf value for word forms, and word length (in characters) on reaction times during reading. The second explored the influence of self-assessed reading habits (reading focus and reading immersion) on reaction times. Both analyses were performed separately for nouns and for verbs, which display markedly different morphological behavior in Czech. This separation was exploratory in nature, but we considered it potentially informative given that nouns and verbs are the most frequent content word classes in the corpus, yet they differ substantially in terms of lemma and word form frequency, as well as surprisal. The average Zipf lemma frequency for verbs was slightly higher (4.31) when compared to nouns (4.03), while nouns show slightly higher surprisal values. These differences may interact with processing measures such as reaction time and thus motivate a separate examination of these two parts of speech.

#### Data analysis

For both analyses, data preprocessing followed these steps: First, we included only participants who took part in both data collection rounds, did not report serious reading difficulties (e.g., dyslexia), and did not report alcohol consumption within the preceding 24 h. This resulted in a final sample of 929 participants (751 female, 168 male, eight nonbinary, and two undisclosed). Second, we excluded words that were part of multiword sequences. Third, we removed all reaction times (RTs) below 100 ms or above 10,000 ms. Reaction times were then log-transformed, with the upper cut-off point set at the mean logRT + 3 standard deviations. In total, 1.43% of RTs were excluded. For the analysis, we used separate subsets for nouns and verbs.

Linear mixed-effects models (Bates et al., 2014; Matuschek et al., 2017) were used for statistical analysis. In both analyses, log-transformed reaction times were used as the dependent variable.

The first analysis examined word length (in characters), Zipf value for lemmas, word surprisal, and Zipf value for word forms. Notably, this analysis was complicated by correlations among some of the variables. The correlation matrix (see Table 6) reveals that Zipf values for lemmas and for word forms are strongly correlated for both nouns ($r=0.9$) and verbs ($r=0.918$). For verbs, in particular, there are moderately strong correlations between word length and Zipf values for lemmas ($r=-0.682$) and word forms ($r=-0.756$). In contrast, word surprisal exhibits only weak correlations with the other measures.

**Table 6:** Pearson correlation coefficients for word form length, Zipf values for lemmas and word forms, and word surprisal for nouns and verbs

Nouns				
	Length	Surprisal	ZipfWordF	ZipfLemmaF
Length	1.000	0.193	-0.450	-0.432
Surprisal	0.193	1.000	-0.357	-0.377
ZipfWordF	-0.450	-0.357	1.000	0.900
ZipfLemmaF	-0.432	-0.377	0.900	1.000
Verbs				
Length	1.000	0.321	-0.756	-0.682
Surprisal	0.321	1.000	-0.408	-0.360
ZipfWordF	-0.756	-0.408	1.000	0.918
ZipfLemmaF	-0.682	-0.360	0.918	1.000

These documented correlations pose challenges for statistical modeling, as models that include all these variables simultaneously are prone to collinearity issues, which can lead to uninterpretable effects. To address this, we opted to compare three independent models. Each model included word surprisal as one fixed effect and one of the three other variables (Zipf value for word forms, Zipf value for lemmas, or word length) as the second fixed effect. Both fixed effects were nested under the effect of data collection round. This modeling choice was motivated by the fact that the two rounds showed apparent baseline differences in reading times, with average RTs for both nouns and verbs being approximately 40 ms longer in round 1 than in round 2, likely due to adaptation or practice effects. Modeling the rounds separately thus allowed us to assess the robustness of effects across sessions without conflating them with these overall reaction time differences.

The three models for each word class were then compared using the Akaike information criterion, AIC (Akaike, 1998). All numerical fixed effects were scaled and centered, and the data collection round was treatment-coded, with round 1 as the reference level. Random effects accounted for variability across lemmas and participants, with no random slopes included. This decision was made to ensure comparability across models in the AIC-based model comparison, as some models failed to converge when random slopes were included, and consistent random-effects structures are required for valid AIC comparisons.

The second analysis examined the role of self-assessed reading habits, which were measured using two variables: reading focus and reading immersion. Since these variables are moderately positively correlated (see Table 7), including both in the same model would result in collinearity issues. To address this, we computed their average, referred to as reading habit.

**Table 7:** Pearson correlation coefficients for reading focus and reading immersion in the two collection rounds

	Round 1		Round 2	
	Reading focus	Reading immersion	Reading focus	Reading immersion
Reading focus	1.000	0.469	1.000	0.563
Reading immersion	0.469	1.000	0.563	1.000

**Table 8:** Results for fixed effects from the linear mixed-effects model with word surprisal and word length (in characters) as fixed effects, and reaction time as the dependent variable

	Nouns				Verbs			
Fixed effect	Estimate	SE	*t* value	*p*	Estimate	SE	*t* value	*p*
Round 2	-0.100	0.001	-165.390	< 0.001	-0.098	0.001	-116.37	< 0.001
Round 1: Surprisal	0.010	0.001	18.910	< 0.001	0.021	0.001	29.56	< 0.001
Round 2: Surprisal	0.015	0.001	26.930	< 0.001	0.022	0.001	30.45	< 0.001
Round 1: Length	0.023	0.001	23.610	< 0.001	0.040	0.001	35.31	< 0.001
Round 2: Length	0.011	0.001	11.440	< 0.001	0.029	0.001	25.49	< 0.001

Models were calculated separately for nouns and verbs

In the analysis of reading habit, we ran linear mixed-effects models with reading habit as a fixed effect in interaction with data collection round and included lemmas (with random slope for reading habit) and participants as random effects. Reading habit was centered to the value 4 (representing the central point of the seven-point scale).

#### Results: Word measures

Altogether, we ran three independent models for nouns and three for verbs. Since running more parallel models increases the probability of a type I error (von der Malsburg and Angele, 2017), a Bonferroni correction was applied, setting the *p* value threshold to 0.017 (0.05/3).

The full model results are provided in the HTML output from an RMarkdown script on OSF website: https://osf.io/jg8ha/. Here, we report only the estimates for fixed effects in each model. Table 8 reports effects from the model containing surprisal and word length, Table 9 reports effects from the model with surprisal and Zipf value for lemma, and Table 10 shows effects from the model with surprisal and Zipf value for word. Across all six models, word surprisal was consistently highly significant in both data collection rounds. The other three variables (i.e., word length, Zipf value for lemmas, and Zipf value for word forms) were also highly significant in the models they were included in.

**Table 9:** Results for fixed effects from the linear mixed-effects model with word surprisal and Zipf value for lemma as fixed effects, and reaction time as the dependent variable

	Nouns				Verbs			
Fixed effect	Estimate	SE	*t* value	*p*	Estimate	SE	*t* value	*p*
Round 2	-0.100	0.001	-165.370	< 0.001	-0.098	0.001	-116.239	< 0.001
Round 1: Surprisal	0.008	0.001	14.720	< 0.001	0.025	0.001	35.164	< 0.001
Round 2: Surprisal	0.015	0.001	26.130	< 0.001	0.026	0.001	35.308	< 0.001
Round 1: ZipfLemmaF	-0.038	0.001	-25.430	< 0.001	-0.033	0.004	-8.205	< 0.001
Round 2: ZipfLemmaF	-0.026	0.001	-17.830	< 0.001	-0.025	0.004	-6.108	< 0.001

Models were calculated separately for nouns and verbs

**Table 10:** Results for fixed effects from the linear mixed-effects model with word surprisal and Zipf value for word as fixed effects, and reaction time as the dependent variable

	Nouns				Verbs			
Fixed effect	Estimate	SE	*t* value	*p*	Estimate	SE	*t* value	*p*
Round 2	-0.100	0.001	-165.370	< 0.001	-0.098	0.001	-116.33	<0.001
Round 1: Surprisal	0.009	0.001	16.150	< 0.001	0.022	0.001	30.23	< 0.001
Round 2: Surprisal	0.015	0.001	27.820	< 0.001	0.023	0.001	31.57	< 0.001
Round 1: ZipfWordF	-0.022	0.001	-23.300	< 0.001	-0.039	0.001	-28.24	< 0.001
Round 2: ZipfWordF	-0.010	0.001	-10.440	< 0.001	-0.030	0.001	-21.32	< 0.001

Models were calculated separately for nouns and verbs

The Akaike information criterion (Akaike, 1998) identified the model containing Zipf value for lemma (see Table 9) as the best-fitting one for nouns and the model containing word length (see Table 8) as the best-fitting one for verbs.

#### Results: Reading habits

The fixed effects from the linear mixed effects models targeting reaction times for nouns and verbs are presented in Table 11.

**Table 11:** Results for fixed effects from the linear mixed-effects model with reading habit and round as fixed effects, and reaction time as the dependent variable

	Nouns				Verbs			
Fixed effect	Estimate	SE	*t* value	*p*	Estimate	SE	*t* value	*p*
Round 2	-0.097	0.001	-139.666	< 0.001	-0.095	0.001	-98.082	< 0.001
Reading habit	0.006	0.001	9.882	< 0.001	0.005	0.001	6.511	< 0.001
Round 2: Reading habit	-0.005	0.001	-8.377	< 0.001	-0.005	0.001	-5.785	< 0.001

Models were calculated separately for nouns and verbs

**Fig. 5:**
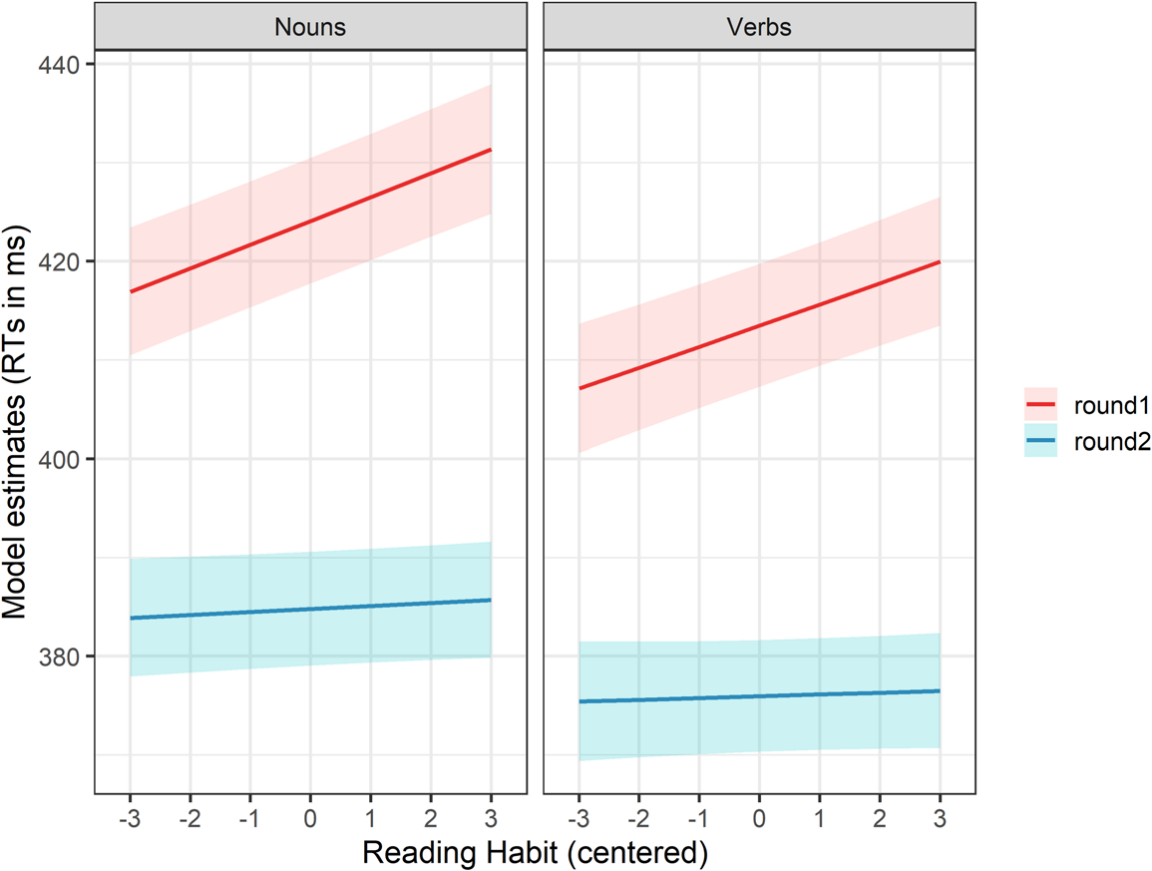
Estimates from the linear mixed-effects models targeting reaction times (as a dependent variable), and the data collection round and reading habit in interaction as the fixed effects. The models were calculated separately for nouns and for verbs

The model revealed an expected effect of data collection round (reaction times being faster in the second data collection round), and a significant positive effect of reading habit (in the first data collection round, participants who assessed themselves as more focused and immersed during reading were reading slower). Interestingly, there was a significant negative interaction between data collection round and reading habit. To further explore this interaction, we plotted the model estimates (i.e., predicted RTs) and the results are presented in Fig. 5.

Interestingly, the interaction shows that the effect of reading habit, which was clearly observable in the first data collection round, has not remained in the second round of data collection. We will return to these findings in the Discussion.

#### Discussion

Across all models, we observed a robust effect of data collection round: participants were significantly faster when completing the experimental task for the second time. This finding is particularly noteworthy given that the interval between the two rounds was approximately 1 month. It suggests that previously reported task adaptation effects in sentence processing (e.g., Chromý and Tomaschek, 2024; Prasad and Linzen, 2021) persist even after a substantial delay. Future research may benefit from linking such effects to adaptation phenomena in other domains, such as visual adaptation (e.g., Webster, 2015) performance adaptation (e.g., Stasielowicz, 2020), or motor learning (e.g., Krakauer et al., 2019; McDougle et al., 2016). Specifically, it would be interesting to investigate whether analogous effects across domains are grounded in similar underlying mechanisms in future research.

For both word classes, we observed consistent effects of Zipf values (used to operationalize word or lemma frequency), word length, and surprisal. These findings are in line with prior research: word frequency and word length are well-established predictors of reading times in written word processing (e.g., Barton et al., 2014; Brysbaert et al., 2018; Raney and Rayner, 1995; White et al., 2018; Yap and Balota, 2009; Hauk and Pulvermüller, 2004; Kříž and Chromý, 2020), with robust effects reported across multiple languages (see Kuperman et al., 2024). Surprisal, likewise, is widely recognized as a predictor of processing difficulty (e.g., Levy, 2008; Demberg and Keller, 2008; Lowder et al., 2018; Shain et al., 2024).

Interestingly, the two word classes differed in which model was identified as the best-fitting one according to the Akaike information criterion (Akaike, 1998). For nouns, the model containing lemma frequency (Zipf values for lemmas) provided the best fit, whereas for verbs, the model with word length in characters was preferred. This finding is potentially interesting and worth deeper analysis in the future. Our preliminary interpretation is that this pattern may be due to differences in the distributions of frequency and length across word classes: while nouns and verbs were relatively similar in length (a mean difference of only 0.18 characters), they differed substantially in frequency (a mean difference of 19.82 words per million; see Table 5.

Our second analysis investigated general reading speed in relation to individual differences in reading strategies or habits. Previous studies (Mak and Willems, 2019; Magyari et al., 2020) have shown that higher mental imagery correlates with slower reading (e.g., longer fixation durations). We focused on self-assessed reading habits, operationalized through two questions: (i) “While reading, to what extent do you focus on how the text is written and how individual formulations change its meaning?” and (ii) “While reading a text, I am typically immersed in all of its details and try to understand it fully.” Responses to these questions were highly correlated, allowing us to use their average (termed reading habit) as a predictor in our models.

Our results revealed a significant positive effect of reading habit, indicating that individuals who focus more on textual details and immerse themselves deeply in comprehension tend to read more slowly. Interestingly, this effect disappeared in the second round of the experiment. We interpret this as evidence of task adaptation: more careful and focused readers became faster once they were familiar with the task and/or stimuli, aligning their performance with readers who self-assess as less detail-oriented and more superficial.

The role of reading habit may relate to previous findings from studies employing reading-aloud protocols (e.g., Pressley and Afflerbach, 2012; Milne, 2021), which highlight individual differences in reading strategies. Admittedly, the operationalization of reading habit in the HeCz corpus is relatively simple – partly because, to our knowledge, no validated measure for this construct currently exists. Nevertheless, even this basic measure, based on just two questions, appears to yield a predictor that significantly influences reaction times.

Altogether, these findings highlight the influence of previously underexplored variables, such as participants’ reading habits, on reading speed. This offers a potentially fruitful direction for further investigation, particularly in understanding how individual differences in cognitive and behavioral reading strategies interact with task demands.

## Conclusion

The HeCz corpus represents a substantial resource for researchers investigating reading processes, both from a coarse-grained and more fine-grained perspective. As the largest behavioral reading corpus to date, it enables to investigate a wide range of other questions related to reading processes. Its comprehensive annotation enables a wide range of research questions to be opened up and addressed, including some that have never been conducted on a dataset of this scale.

## Open Practices Statement

The data and materials are available at Open Science Framework: https://osf.io/jg8ha/.

## Data Availability

The HeCz corpus is fully available on OSF website: https://osf.io/jg8ha/. It is organized into four separate csv files. Comprehensive documentation to each file is also provided.

## Declarations

### Code availability

The R script used for the analysis in this paper is also available on OSF website: https://osf.io/jg8ha/ together with an html output of RMarkdown.
